# Enhanced anti-tumor and anti-angiogenic effects of metronomic cyclophosphamide combined with Endostar in a xenograft model of human lung cancer

**DOI:** 10.3892/or.2012.1828

**Published:** 2012-05-22

**Authors:** RUI WANG, SHUKUI QIN, YUQING CHEN, YUMEI LI, CHANGJIE CHEN, ZISHU WANG, RONGSHENG ZHENG, QIONG WU

**Affiliations:** 1 Department of Medical Oncology, The Affiliated Hospital of Bengbu Medical College, Bengbu, Anhui 233004; 2 Department of Respiratory Diseases, The Affiliated Hospital of Bengbu Medical College, Bengbu, Anhui 233004; 3 Department of Bioscience, Bengbu Medical College, Bengbu, Anhui 233004; 4 Department of Medical Oncology, PLA Cancer Center, Nanjing Bayi Hospital, Nanjing, Jiangsu 210002, P.R. China

**Keywords:** non-small cell lung cancer, metronomic chemotherapy, cyclophosphamide, Endostar, angiogenesis

## Abstract

Standard chemotherapy for advanced NSCLC has reached a therapeutic plateau. More effective strategies must be explored. The purpose of this study was to evaluate the role of metronomic chemotherapy combined with an angiogenesis inhibitor in non-small cell lung cancer (NSCLC). A total of 114 BALB/c nude mice were inoculated subcutaneously with human NSCLC cells (A549), and when xenograft tumors were palpable, mice were randomly injected with saline as controls (Ctrl), or treated with metronomic cyclophosphamide (MET CPA), recombinant human endostatin, Endostar (Endo), MET CPA combined with Endostar (MET CPA + Endo) or maximum tolerance dose of CPA (MTD CPA), respectively. The growth of xenograft tumors and mouse survival were monitored. The frequency of peripheral blood circulating endothelial cells (CECs), microvessel density (MVD) and pericyte coverage was determined using flow cytometry and immunofluorescence staining. In comparison with the controls, treatment with either drug significantly inhibited the growth of xenograft tumors in mice. Treatment with MET CPA or Endostar, but not with MTD CPA, significantly reduced the frequency of peripheral blood total and viable CECs and the value of MVD. Endostar also considerably reduced pericyte coverage in xenograft tumors. Moreover, MET CPA combined with Endostar further reduced the frequency of peripheral blood CECs, the value of MVD, and pericyte coverage, with concomitant delay in tumor growth and extension of mouse survival. Our results indicate that MET CPA combined with Endostar results in enhanced anti-tumor and anti-angiogenic effects in a xenograft model of human lung cancer. Combined therapy with metronomic chemotherapy and an angiogenesis inhibitor may serve as a promising treatment strategy for patients with advanced NSCLC.

## Introduction

Lung cancer is a leading cause of cancer-related mortality worldwide, and ~80% of cases with lung cancer are non-small cell lung cancer (NSCLC) (1,2). The majority of NSCLC patients present with advanced disease at diagnosis. Cytotoxic chemotherapy, specifically, platinum-based doublets, has been recommended as standard treatment for these patients (3). However, standard chemotherapy for advanced NSCLC has reached a therapeutic plateau with a median survival of ~1 year (4,5). Therefore, more effective strategies must be explored.

Metronomic chemotherapy (MET) is a therapeutic approach by chronic administration of chemotherapeutic agents at a relatively low and minimally toxic dose without a prolonged drug-free break (6). During the past decade, MET with different chemotherapeutic agents has been demonstrated to significantly reduce side effects associated with standard chemotherapy, and to inhibit tumor growth and metastasis by antagonizing angiogenesis (6,7), a hallmark event during cancer development and a key component for the continuous growth and metastasis of tumor cells. Additionally, recent studies have suggested that MET may be a multi-targeted anti-tumor strategy by restoring anti-tumor immunity and inducing tumor dormancy (7). A previous study has shown that metronomic administration of cyclophosphamide (CPA) in drinking water at low doses (10–40 mg/kg) on a daily basis is effective in delaying the growth of orthotopic breast or ectopic colon cancer xenografts in nude or SCID mice (8). This study, together with many preclinical experiments and clinical trials, provides accumulative evidence that MET can maintain the therapeutic response, minimize the relapse after conventional chemotherapy, and overcome the resistance (6,7,9).

Endostar is a recombinant human endostatin with an additional nine-amino acid sequence at the N-terminal of the protein to help in protein purification, solubility and stability (10). This anti-angiogenic drug is used in combination with standard chemotherapy for the treatment of advanced NSCLC in China and being investigated in other types of cancer, including breast, colon and pancreatic cancers (11). However, whether Endostar combined with MET CPA could enhance anti-tumor and anti-angiogenic effects in advanced NSCLC remains unclear. In the present study, we employed a xenograft model of human NSCLC to evaluate the role of MET CPA and/or Endostar on the growth and angiogenesis of implanted lung cancers as well as survival of tumor-bearing animals.

## Materials and methods

### Cell culture and chemicals

A human lung adenocarcinoma cell line, A549, was purchased from the Chinese Academy of Sciences (Shanghai, China) and cultured in Dulbecco’s modified Eagle’s medium (DMEM, Gibco BRL, Carlsbad, CA, USA) supplemented with 10% fetal bovine serum (FBS, Gibco) at 37°C in a humidified atmosphere containing 5% CO_2_. Endostar was obtained from Simcere Pharmaceutical Group (Nanjing, China) and cyclophosphamide monohydrate (CPA) was purchased from International Laboratory (San Bruno, CA, USA).

### Mouse xenograft model and treatments

The experimental protocols were approved by the Institutional Animal Care and Use Committees of Bengbu Medical College (Anhui, China). Four-week-old BALB/c nude mice were purchased from the Experimental Animal Center, Chinese Academy of Sciences (Shanghai, China). Mice were housed (5 mice/per cage) in a specific pathogen-free facility at a constant temperature of 25–27°C, a constant humidity of 40–50% on a cycle of 12/12-h light/dark, with access to autoclaved food and water *ad libitum*. To establish the xenograft model, A549 cells (2.5×10^6^ in 100 μl of DMEM) were injected subcutaneously into the back close to the right axilla of individual animals. The tumor growth was monitored every 3 days by measuring the length (L) and width (W) of the tumors, with the volume (V) calculated as V = 0.52 × L × W^2^, as previously described (12). When the tumors grew to ~200 mm^3^, the tumor-bearing mice were randomly assigned and injected intraperitoneally (i.p.) with saline vehicle daily as the control group (Ctrl), or treated with 10 mg/kg body weight (bw) of CPA daily by gavage as the metronomic CPA group (MET CPA) (8,12), or 4 mg/kg bw of Endostar i.p. daily as the Endostar group (Endo), or the same routes and doses of metronomic CPA and Endostar as the MET CPA combined with Endostar group (MET CPA + Endo), or 100 mg/kg bw of CPA i.p. on days 1, 3 and 5, and repeated every 21 days as the maximum tolerance dose group (MTD CPA), respectively (n=14–15 per group). The growth of xenograft tumors was monitored up to 12 weeks post-treatment, and survival of mice was recorded. Eight mice from individual groups were sacrificed randomly at 9 weeks post-treatment, with the blood samples collected and the tumor tissues dissected out for further analysis.

### Flow cytometry analysis of peripheral blood circulating endothelial cells (CECs)

The frequency of peripheral blood CECs was determined using four-color flow cytometry (FACSCalibur, BD Biosciences, San Jose, CA), as described previously (13,14). Briefly, peripheral blood nuclear cells were prepared from individual mice after lyzing red blood cells. Subsequently, the cells were stained with APC-anti-CD45 (1:10, Biolegend, San Diego, CA, USA) to exclude hematopoietic cells, PE-anti-CD146 (1:10, Biolegend), and 7-amino-actinomycin D (7AAD, 1:10, Biolegend) to distinguish viable CECs from dead ones. The APC-rat-IgG2b (1:10, Biolegend) and PE-rat-IgG2a (1:10, Biolegend) were used as isotype controls. A total of 100,000 events from individual samples were gated on R1 to exclude platelets, dead cells, and debris and the CD45^−^CD146^+^ cells (R2) were further analyzed for their 7AAD staining. As a result, CD146^+^7AAD^+^ cells represent apoptotic CECs while CD146^+^7AAD^−^ cells represent viable ones.

### Immunofluorescence staining

The MVD and pericyte coverage in xenograft tumors were quantified using a laser confocal microscope (Nikon, Japan) (12). Briefly, tumor tissues from individual mice at 9 weeks post-treatment were fixed in 10% zinc-formalin for 1 h at 4°C, and dehydrated in 30% sucrose in PBS until the tissues sank to the bottom. The tumor tissues were embedded in optimal cutting temperature (OCT) medium, frozen and sectioned. At least 20 tissue sections at 4 μm were prepared for individual tumors. Five inconsecutive sections from individual tumors were selected for immunofluorescence staining. Individual sections were stained with monoclonal antibodies against CD31 (1:50, Biolegend), NG2 (1:50, Abcam, Cambridge, UK) or isotype controls at 4°C overnight. After being washed with PBS 3 times (10 min each), the tissue sections were probed with fluorescent-labeled secondary antibodies (1:100, Abcam) at 37°C for 1 h. The sections were mounted with mounting buffer containing 4′,6-diamidino-2-phenylindole (DAPI, Santa Cruz, CA, USA) and covered with a coverslip. The value of MVD was counted as the number of CD31^+^ tubular structures from five random fields and pericyte coverage was quantified as the percent of NG2^+^ signals among circumference of CD31^+^ cross-sections or length of CD31^+^ longitudinal-sections from five vessel profiles under high-power field (magnification ×400) per section.

### Statistical analysis

Statistical analysis was performed with SPSS 11.0 software. All measurements were presented as mean ± SD. Statistical comparisons were first tested for homogeneity of variances. Multiple comparisons were performed using One-way ANOVA analysis, with two-two comparisons using Student-Newman-Keul (SNK) test. Survival analysis was performed with the log-rank test. P<0.05 was considered statistically significant.

## Results

### MET CPA combined with Endostar inhibits the growth of xenograft tumors in vivo

**Figure 1:**
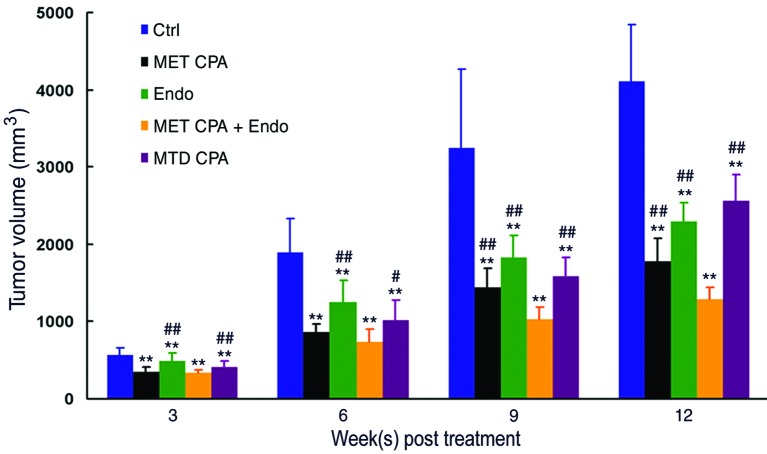
MET CPA combined with Endostar inhibits the growth of xenograft tumors in mice. BALB/c nude mice were inoculated subcutaneously with human NSCLC A549 cells and when a palpable tumor developed, mice were randomly assigned into five groups (n=15 for the Ctrl, MET CPA, Endo, MET CPA + Endo groups; n=14 for the MTD CPA group). The tumor volumes of different groups were compared at indicated time-points. Data shown are mean ± SD of tumor volumes in individual groups of mice. ^**^P<0.01 vs. the Ctrl group; ^#^P<0.05, ^##^P<0.01 vs. the MET CPA + Endo group.

The growth of xenograft tumors in mice treated with MET CPA alone, Endostar alone, MET CPA plus Endostar, or MTD CPA alone is shown in Fig. 1. In comparison with that in the controls, the tumor volumes in all drug-treated groups of mice were significantly smaller (P<0.01), indicating that treatment with either drug inhibited the growth of xenograft tumors *in vivo*. More importantly, the tumor volumes in the MET CPA + Endo group of mice were significantly less than that in all other groups at 3, 6, 9 and 12 weeks post-treatment, except that the difference between the MET CPA and MET CPA + Endo group was not significant at 3 and 6 weeks post-treatment (P>0.05). Collectively, these data indicated that treatment with either drug inhibited the growth of xenograft tumors, and that MET CPA combined with Endostar had the most robust anti-tumor effect in this experimental model.

### MET CPA combined with Endostar reduces the frequency of peripheral blood CECs in the tumor-bearing mice

**Figure 2:**
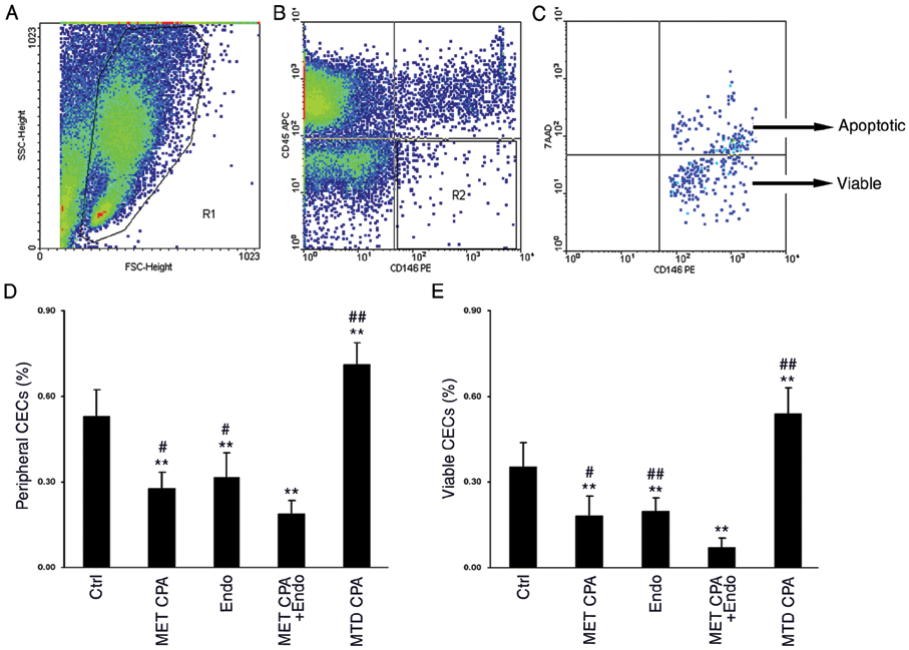
MET CPA combined with Endostar decreases the frequency of peripheral blood total and viable CECs. The frequency of peripheral blood total and viable CECs in individual mice was determined by flow cytometry analysis. Peripheral blood nuclear cells were stained with APC-anti-CD45, PE-anti-CD146 and 7AAD. (A) The cells were gated on FSC vs. SSC plot. (B) The CD45^−^CD146^+^ CECs were selected. (C) To distinguish 7AAD^+^ apoptotic CECs from 7AAD^−^ viable ones. The frequency of peripheral blood total (D) and viable CECs (E) in individual groups of mice was quantitatively analyzed. Data are expressed as mean ± SD of the frequency of peripheral blood CECs in individual groups (n=8 per group). ^**^P<0.01 vs. the Ctrl group; ^#^P<0.05, ^##^P<0.01 vs. the MET CPA + Endo group.

The peripheral blood CECs is an excellent surrogate marker for evaluation of vascular damage and neoangiogenesis, which are involved in the growth and metastasis of cancer (15,16). To understand whether alterations in vascular damage or angiogenesis are involved in the delayed growth of xenograft tumors conferred by MET CPA combined with Endostar, we first characterized the frequency of peripheral blood CECs from mice of different groups at 9 weeks post-treatment (Fig. 2). First, there was no significant difference in the total numbers of blood nuclear cells among these groups of mice (data not shown). Second, relatively high frequency of total and viable peripheral blood CECs (0.53±0.09 and 0.35±0.08%, respectively) was detected in the Ctrl group. Third, while treatment with MTD CPA increased the frequency of both total and viable CECs in mice (0.71±0.07 and 0.54±0.09%, respectively, P<0.01), treatment with MET CPA or Endostar significantly reduced it (P<0.01). Furthermore, the frequency of both total and viable CECs in the MET CPA + Endo group of mice (0.19±0.05 and 0.07±0.03%, respectively) was significantly lower than that in the other groups of mice (all P<0.05). Collectively, these data indicated that MET CPA or Endostar significantly decreased the frequency of peripheral blood CECs and treatment with both further reduced it in mice.

### MET CPA combined with Endostar reduces the tumor-associated MVD

**Figure 3:**
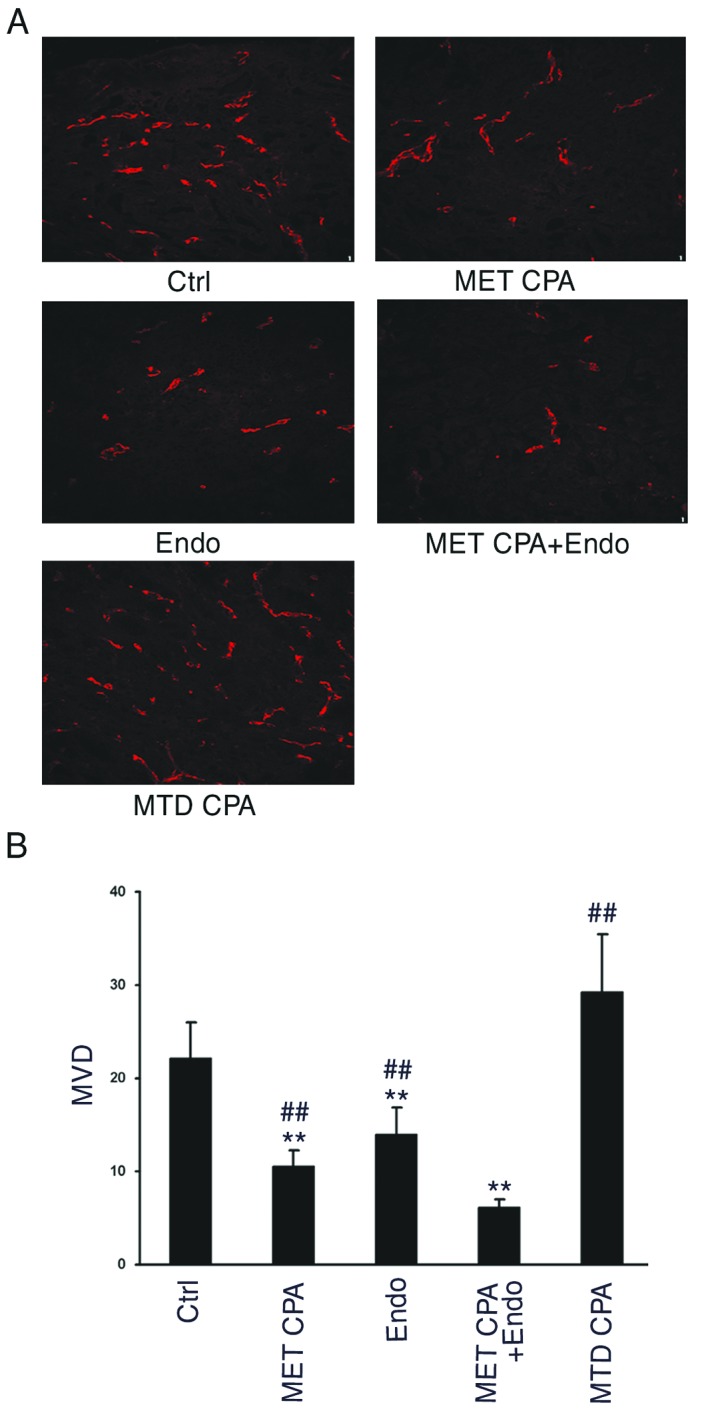
MET CPA combined with Endostar reduces tumor MVD. The MVD in xenograft tumors from different groups of mice was determined by immunofluorescence staining with anti-CD31 antibody. (A) Representative images of immunofluorescence staining of endothelial cells in tumor tissues from different groups of mice. (B) Quantifications of MVD. Data are expressed as mean ± SD of the MVD value of individual groups (n=8 per gorup). ^**^P<0.01 vs. the Ctrl group; ^##^P<0.01 vs. the MET CPA + Endo group.

Next, we examined MVD in xenograft tumors by immunofluorescence staining with antibody against CD31, a specific marker of endothelial cells. As shown in Fig. 3, a high level of MVD was detected in tumor tissues from the Ctrl and MTD CPA groups of mice, but significantly lower value of MVD was observed in tumor tissues from the MET CPA, Endo, and MET CPA + Endo groups of mice. Quantitative analysis indicated that the value of MVD in the Ctrl group (22.08±3.98) was similar to that in the MTD CPA group of mice (29.29±6.11, P>0.05), both significantly greater than that in the MET CPA (10.58±1.71) and Endo groups (14.04±2.86, P<0.01). These data suggested that MET CPA or Endostar, but not MTD CPA, significantly inhibited the formation of microvascular vessels in xenograft tumors. Notably, the value of MVD in tumor tissues from the MET CPA + Endo group of mice (6.08±0.96) was not only significantly less than that in the Ctrl and MTD CPA groups of mice, but also significantly less than that in the MET CPA or Endo groups (P<0.01 vs. other groups). Thus, MET CPA plus Endostar could further mitigate the tumor growth-induced microvassel angiogenesis in mice.

### MET CPA combined with Endostar reduces pericyte coverage in xenograft tumors

**Figure 4:**
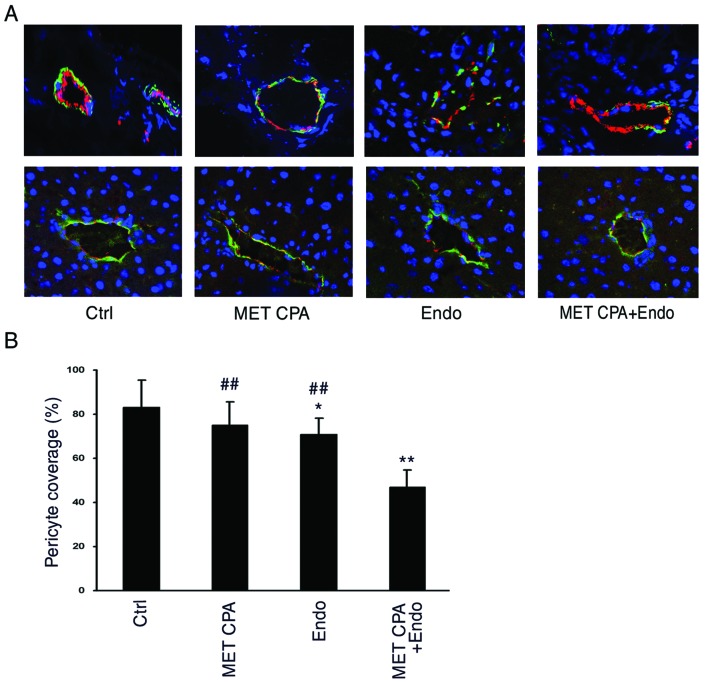
MET CPA combined with Endostar reduces pericyte coverage in xenograft tumors but not in livers. The pericyte coverage in xenograft tumors and livers from individual groups of mice was characterized by immunofluorescence staining with anti-NG2 antibody (green), anti-CD31 antibody (red) and DAPI (blue). (A) Representative images of microvessels in the tumors (top panels) and livers (lower panels) from different groups of mice. (B) Quantifications of pericyte coverage in the tumors from different groups of mice. Data are expressed as mean ± SD of the percent of pericyte coverage in individual groups (n=8 per group). There was no significant difference in pericyte coverage in livers of different groups of mice (data not shown). ^*^P<0.05, ^**^P<0.01 vs. the Ctrl group; ^##^P<0.01 vs. the MET CPA + Endo group.

Given that pericytes can modulate multiple behaviors of endothelial cells, we assessed pericyte coverage as a measure of tumor angiogenesis by immunofluorescence staining of tumor tissue sections with antibody against NG2, a specific marker of pericytes. As illustrated in Fig. 4, the value of pericyte coverage was 83.26±12.26% in the Ctrl group of mice, similar to that detected in the MET CPA group of mice (75.10±10.71%), indicating that treatment with MET CPA alone did not dramatically alter pericyte coverage (P>0.05, Fig. 4A, upper panels and B). However, the value of pericyte coverage in the Endo group of mice (71.16±7.31%) was significantly less than that in the Ctrl group (P<0.05). More interestingly, the value of pericyte coverage in the MET CPA + Endo group of mice was further reduced to 46.77±7.66%, as compared with that in other groups (P<0.01). In contrast, the dissociation of pericytes from endothelial cells and the differences in pericyte coverage from different treatments were not detected in livers of mice (Fig. 4A, lower panels), suggesting that anti-angiogenic effect only occurred in the tumor but not in normal tissue. These data further indicate that MET CPA plus Endostar inhibits neoangiogenesis in the tumors, which may contribute to the inhibition of tumor growth in mice.

### MET CPA combined with Endostar prolongs survival of tumor-bearing mice

**Figure 5:**
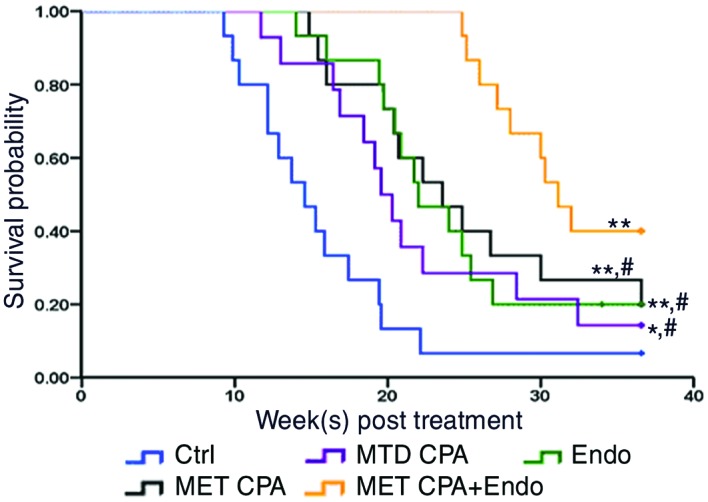
MET CPA combined with Endostar prolonges survival of tumor-bearing mice. Following inoculation with the tumor, survival of individual mice was monitored up to 37 weeks after treatment. Data are shown as survival probability and the difference among groups was determined by log-rank test (n=15 for the control, MET CPA, Endo, MET CPA + Endo groups; n=14 for the MTD CPA group). ^*^P<0.05, ^**^P<0.01 vs. the Ctrl group; ^#^P<0.05 vs. the MET CPA + Endo group.

Finally, we examined the impact of different treatments on survival of tumor-bearing mice. Consistent with the alterations in tumor burden, mice in the Ctrl group survived a shorter period with a median survival time of 14.57 weeks (Fig. 5). In contrast, mice in the MET CPA, Endo, and MTD CPA groups survived significantly longer with a median survival time of 23.57, 22.00 and 19.56 weeks, respectively (P<0.05 vs. control group). More importantly, mice in the MET CPA + Endo group survived much longer with a median time of 31.14 weeks (P<0.05 vs. other groups). These data clearly demonstrated that MET CPA combined with Endostar further prolonged survival of tumor-bearing mice.

## Discussion

Here we report that the combination of MET CPA and Endostar exhibits enhanced anti-tumor effects with respect to the growth of xenograft tumors and survival of tumor-bearing mice. Mechanistically, combined treatment further reduced the frequency of total and viable peripheral blood CECs, the value of MVD, and pericyte coverages in xenograft tumors derived from NSCLC. The activities on tumor-associated angiogenesis may contribute to anti-tumor effects as conferred by MET CPA combined with Endostar.

Angiogenesis plays a critical role in continuous tumor growth and tumor dissemination, involving in extensive crosstalk among tumor cells, vascular endothelial cells, stromal cells and pericytes (17). This process is associated with the alterations on the cellular level both locally within the tumor and systematically in the blood circulation. It starts with pericyte-endothelial cell dissociation, followed by proliferation and invasion of endothelial cells, formation of endothelial tubulogenesis and vascular stabilization (18). Consequently, an increased MVD and pericyte coverage are often observed in tumors with a high level of angiogenesis and correlated with unfavorable clinicopathologic parameters (19). Systemically, at least two distinct populations of CECs have been identified: bone marrow-derived circulating endothelial progenitor cells (CEPs, CD45^−^CD146^+^CD34^+^CD133^+^), and mature CECs (CD45^−^CD146^+^CD34^+^CD133^−^) (20). They constitute a rare population of circulating blood cells under physiological condition, but they dramatically increase in response to vascular damage in pathological situations. The frequency of peripheral blood CECs has been considered a useful surrogate marker for angiogenesis in tumor progression (21,22). Furthermore, growing evidence suggests that alterations in the frequency of peripheral blood CECs and their viability are correlated with the therapeutic responses to angiogenesis inhibitor in cancer patients (23–25). Therefore, measurements of peripheral blood CECs, together with MVD and pericyte coverage in the tumors, are valuable for uncovering the role of anti-angiogenic drugs.

In the present study, we examined the impact of MET CPA and/or Endostar on the peripheral blood CECs, MVD and pericyte coverage in tumor-bearing mice. We found that in the control group, peripheral CECs constituted ~0.50% of circulating blood cells, nearly 70% of which were viable CECs, and concomitantly, higher levels of MVD and pericyte coverage were detected in xenograft tumors. In contrast, MET CPA or Endostar significantly reduced the frequency of peripheral blood total and viable CECs and the value of MVD; Endostar also considerably inhibited pericyte coverage in xenograft tumors. More importantly, MET CPA combined with Endostar further reduced the frequency of peripheral blood total and viable CECs, the value of MVD, and pericyte coverage. These data indicate that the combination of MET CAP with Endostar lead to more potent inhibition of neoangiogenesis in the tumors, which may contribute to the inhibition of tumor growth in mice. Interestingly, when we examined pericyte coverage in liver tissues form tumor-bearing mice we found that there was no significant difference in pericyte coverage among the different treatment groups, suggesting that MET CPA or Endostar predominantly affects angiogenesis in tumor tissues. In addition, the levels of angiogenesis in xenograft tumors appear to be negatively associated with the length of survival periods in different groups of mice.

Notably, we found that treatment with MTD CPA effectively inhibited the growth of xenograft tumors, but enhanced peripheral blood CECs in mice, which is consistent with the role of MTD cytotoxic chemotherapy in inducing endothelial damage (26). By contrast, when administered on a metronomic schedule, CPA not only inhibited the growth of tumors, but also reduced the frequency of peripheral blood CECs in mice, with a further reduced level when combined with Endostar. The increased level of CECs may contribute to the repair of damaged vasculature after MTD chemotherapy and the decreased level of CECs suppress the repair and recovery of tumor vasculature which is indispensable to tumor growth and metastasis. As to the underlying mechanism for the opposite effects of MTD CPA and MET CPA on CECs level, a possible explanation is their inverse effects on the mobilization and viability of CEPs. Mice treated with MTD CPA experienced a robust CEPs mobilization a few days after the end of drug administration, while the CEPs numbers and viability in mice treated with MET CPA was sustained at a very low level for a much prolonged period (13,14).

Endostar has been demonstrated to antagonize the VEGF-mediated signaling in vascular endothelial cells (27), and in these cells, MET CPA markedly increases the level of thrombospondin 1 (28), which is a well known endogenous inhibitor of angiogenesis. The combination of Endostar and MET CPA may inhibit the angiogenesis and growth of tumors. Indeed, in the present study, the combination of MET CPA and Endostar resulted in robust anti-tumor effects through enhanced inhibition of tumor-associated angiogenesis. Our data are consistent with findings from other studies (12,23), and support the notion that MET chemotherapy combined with an angiogenesis inhibitor is a better strategy for the treatment of cancers (29). Conceivably, this therapeutic strategy can be moved from bench to bedside, particularly for a maintenance therapy after efficient first-line chemotherapy on patients with advanced NSCLC, to achieve sustainable tumor control and a longer progression-free survival (30).

In conclusion, our results indicate that MET CPA combined with Endostar results in enhanced anti-tumor and anti-angiogenic effects in a xenograft model of human lung cancer. These findings may aid in the design of clinical trials to investigate the efficacy of metronomic chemotherapy combined with an angiogenesis inhibitor for patients with advanced NSCLC, which may serve as a promising treatment strategy.

## References

[1] Jemal A, Bray F, Center MM, Ferlay J, Ward E, Forman D (2011). Global cancer statistics. CA Cancer J Clin.

[2] Parkin DM, Bray F, Ferlay J, Pisani P (2005). Global cancer statistics, 2002. CA Cancer J Clin.

[3] Goffin J, Lacchetti C, Ellis PM, Ung YC, Evans WK (2010). First-line systemic chemotherapy in the treatment of advanced non-small cell lung cancer: a systematic review. J Thorac Oncol.

[4] Felip E, Cedrés S, Checa E, Martinez P (2010). How to integrate current knowledge in selecting patients for first line in NSCLC?. Ann Oncol.

[5] Thatcher N, Heighway J (2010). Maintenance and consolidation therapy in patients with unresectable stage III/IV non-small cell lung cancer. Oncologist.

[6] Kerbel RS, Kamen BA (2004). The anti-angiogenic basis of metronomic chemotherapy. Nat Rev Cancer.

[7] Pasquier E, Kavallaris M, Andre N (2010). Metronomic chemotherapy: new rationale for new directions. Nat Rev Clin Oncol.

[8] Man S, Bocci G, Francia G, Green SK, Jothy S, Hanahan D, Bohlen P, Hicklin DJ, Bergers G, Kerbel RS (2002). Antitumor effects in mice of low-dose (metronomic) cyclophosphamide administered continuously through the drinking water. Cancer Res.

[9] Kong DS, Lee JI, Kim WS, Son MJ, Lim do H, Kim ST, Park K, Kim JH, Eoh W, Nam DH (2006). A pilot study of metronomic temozolomide treatment in patients with recurrent temozolomide-refractory glioblastoma. Oncol Rep.

[10] Song HF, Liu XW, Zhang HN, Zhu BZ, Yuan SJ, Liu SY, Tang ZM (2005). Pharmacokinetics of His-tag recombinant human endostatin in Rhesus monkeys. Acta Pharmacol Sin.

[11] Jia H, Kling J (2006). China offers alternative gateway for experimental drugs. Nat Biotechnol.

[12] Pietras K, Hanahan D (2005). A multitargeted, metronomic, and maximum-tolerated dose ‘chemo-switch’ regimen is antiangiogenic, producing objective responses and survival benefit in a mouse model of cancer. J Clin Oncol.

[13] Bertolini F, Paul S, Mancuso P, Monestiroli S, Gobbi A, Shaked Y, Kerbel RS (2003). Maximum tolerable dose and low-dose metronomic chemotherapy have opposite effects on the mobilization and viability of circulating endothelial progenitor cells. Cancer Res.

[14] Goon PK, Lip GY, Stonelake PS, Blann AD (2009). Circulating endothelial cells and circulating progenitor cells in breast cancer: relationship to endothelial damage/dysfunction/apoptosis, clinicopathologic factors, and the Nottingham Prognostic Index. Neoplasia.

[15] Goon PK, Lip GY, Boos CJ, Stonelake PS, Blann AD (2006). Circulating endothelial cells, endothelial progenitor cells, and endothelial microparticles in cancer. Neoplasia.

[16] Mancuso P, Calleri A, Cassi C, Gobbi A, Capillo M, Pruneri G, Martinelli G, Bertolini F (2003). Circulating endothelial cells as a novel marker of angiogenesis. Adv Exp Med Biol.

[17] Folkman J (2007). Angiogenesis: an organizing principle for drug discovery?. Nat Rev Drug Discov.

[18] Raza A, Franklin MJ, Dudek AZ (2010). Pericytes and vessel maturation during tumor angiogenesis and metastasis. Am J Hematol.

[19] Miyata Y, Kanda S, Ohba K, Nomata K, Hayashida Y, Eguchi J, Hayashi T, Kanetake H (2006). Lymphangiogenesis and angiogenesis in bladder cancer: prognostic implications and regulation by vascular endothelial growth factors-A, -C, and -D. Clin Cancer Res.

[20] Mariucci S, Rovati B, Bencardino K, Manzoni M, Danova M (2008). Flow cytometric detection of circulating endothelial cells and endothelial progenitor cells in healthy subjects. Int J Lab Hematol.

[21] Martin-Padura I, Bertolini F (2009). Circulating endothelial cells as biomarkers for angiogenesis in tumor progression. Front Biosci (Schol Ed).

[22] Bertolini F, Shaked Y, Mancuso P, Kerbel RS (2006). The multifaceted circulating endothelial cell in cancer: towards marker and target identification. Nat Rev Cancer.

[23] Dellapasqua S, Bertolini F, Bagnardi V, Campagnoli E, Scarano E, Torrisi R, Shaked Y, Mancuso P, Goldhirsch A, Rocca A, Pietri E, Colleoni M (2008). Metronomic cyclophosphamide and capecitabine combined with bevacizumab in advanced breast cancer. J Clin Oncol.

[24] Furstenberger G, von Moos R, Lucas R, Thurlimann B, Senn HJ, Hamacher J, Boneberg EM (2006). Circulating endothelial cells and angiogenic serum factors during neoadjuvant chemotherapy of primary breast cancer. Br J Cancer.

[25] Zhang H, Vakil V, Braunstein M, Smith EL, Maroney J, Chen L, Dai K, Berenson JR, Hussain MM, Klueppelberg U, Norin AJ, Akman HO, Ozçelik T, Batuman OA (2005). Circulating endothelial progenitor cells in multiple myeloma: implications and significance. Blood.

[26] Laquente B, Vinals F, Germa JR (2007). Metronomic chemotherapy: an antiangiogenic scheduling. Clin Transl Oncol.

[27] Ling Y, Yang Y, Lu N, You QD, Wang S, Gao Y, Chen Y, Guo QL (2007). Endostar, a novel recombinant human endostatin, exerts antiangiogenic effect via blocking VEGF-induced tyrosine phosphorylation of KDR/Flk-1 of endothelial cells. Biochem Biophys Res Commun.

[28] Bocci G, Francia G, Man S, Lawler J, Kerbel RS (2003). Thrombospondin 1, a mediator of the antiangiogenic effects of low-dose metronomic chemotherapy. Proc Natl Acad Sci USA.

[29] Murray A, Little SJ, Stanley P, Maraveyas A, Cawkwell L (2010). Sorafenib enhances the in vitro anti-endothelial effects of low dose (metronomic) chemotherapy. Oncol Rep.

[30] Fidias P, Novello S (2010). Strategies for prolonged therapy in patients with advanced non-small-cell lung cancer. J Clin Oncol.

